# Acne and smoking: is there a relationship?

**DOI:** 10.1186/1471-5945-5-2

**Published:** 2005-03-24

**Authors:** Alireza Firooz, Reza Sarhangnejad, Seyyed Massoud Davoudi, Mansour Nassiri-Kashani

**Affiliations:** 1 Center for Research & Training in Skin Diseases & Leprosy, Tehran University of Medical Sciences, 79 Taleghani Avenue, Tehran, Iran; 2 Department of Dermatology, Baghiat-Allah University of Medical Sciences, Mollasadra Avenue, Tehran, Iran

## Abstract

**Background:**

There are contradictory reports on the relationship between acne vulgaris and cigarette smoking. The objective of this study was to examine the relation between acne and cigarette smoking in a case-control study.

**Methods:**

A questionnaire on smoking habits was offered to 350 patients with acne vulgaris and 350 patients suffering from skin diseases other than acne, aged 15 – 40 years, attending in a skin clinic in Tehran, Iran. The patients completed the questionnaires anonymously in the waiting room.

**Results:**

Two hundred and ninety-three patients with acne (response rate 83.7 %) and 301 patients with other skin diseases (response rate 86.0 %) completed the questionnaires. Twelve acne patients (4.1 %) and 27 control patients (9.0 %) were current smokers (odds ratio = 0.43, 95% confidence limits 0.22 – 0.87, p < 0.05). But after adjustment for sex, this difference was not significant (odds ratio: 0.61, 95% CI: 0.30–1.26, p > 0.05, Mantel-Haenszel test).

**Conclusion:**

An association between acne and cigarette smoking was not found in this study.

## Background

Acne vulgaris is a common, chronic inflammatory disease of pilosebaceous follicles. Several factors have been suggested to influence acne including diet, menstruation, sweating, UV radiation, stress, and occupation [1]. Although some studies have shown that cigarette smoking aggravate acne [2,3], others did not confirm this association [4], or even showed a protective effect [5]. Therefore this study was undertaken to evaluate the relationship between acne vulgaris and cigarette smoking.

## Methods

Three hundred and fifty patients with acne vulgaris and a similar number of patients with skin diseases other than acne, aged 15 – 40 years, attending in a skin clinic in Tehran, Iran were asked to complete a questionnaire on smoking habits anonymously in the waiting room. Acne was diagnosed on a clinical basis by the presence of either of comedones, papules, pustules, nodules or cysts but those with only acne scars and no active lesions were not included.

Current smoking was defined as smoking at least one cigarette in a week. All data were analyzed with the SPSS for windows (release 11.5).

## Results

Two hundred and ninety-three patients with acne and 301 patients with other skin diseases completed the questionnaires (response rates of 83.7% and 86.0%, respectively). Twelve acne patients (4.1%) and 27 controls (9.0%) were current smokers (odds ratio: 0.43, 95% CI: 0.22 – 0.87, p < 0.05). But after adjustment for sex, this difference was not significant (odds ratio: 0.61, 95% CI: 0.30–1.26, p > 0.05, Mantel-Haenszel test). Assuming a 2-sided significance level of 0.05, this study had a power of 77.8% to detect an odds ratio of 2.

The mean age of the patients in the acne group was 22.08 years (SD = 4.60) and in the control group was 26.09 (SD = 6.52) years (t test, p > 0.05). There were 9 smokers among 64 males with acne and 20 smokers among 116 without it (odds ratio: 0.79, 95% CI: 0.33–1.84, p > 0.05). Also there were 3 smokers among 229 females with acne and 7 smokers among 185 without it (odds ratio: 0.34, 95% CI: 0.09–1.32, p > 0.05). Also the amount and duration of smoking were not significantly different between two groups (table 1, Fisher's exact test: p > 0.05).

**Table 1:** The amount and duration of smoking in acne and control patients

Smoking habit		Acne patients (12)	Control patients (27)
Amount	1 cigarette per week	2	5
	1–6 cigarettes per week	2	9
	> 6 cigarettes per week	8	12
	Not mentioned	0	1

Duration	< 3 months	1	2
	3 months – 3 years	6	7
	> 3 years	5	16
	Not mentioned	0	2

## Discussion

We could not find any association between acne and smoking in this study. Although patients with acne were less likely to smoke, but this association was not present after adjustment for sex. The patients included in this study had a wide age range (i.e., 15 to 40 years). As a consequence we could not separate factors associated with acne onset from changes in personal habits occurred during the course of the disease.

Mills for the first time studied the association between acne and smoking [5]. He studied 156 patients with acne through a questionnaire on smoking habits and found that 19.7% of 96 male and 12.1% of female patients were smokers, which was significantly less than national statistics (34.5% and 32.7%, respectively). So he suggested that some component of cigarette, possibly nicotine, has an anti-inflammatory action on acne. We compared the acne patients with patients with other skin diseases attending the same clinic in the same time period, and believe that they are more suitable as a control group that national statistics. Also Mills studied patients with severe acne under treatment with isotretinoin, whereas this study included patients with all forms of acne.

Jemec et al [4] studied the prevalence of acne and the smoking habits of a random sample of 186 15- to 22-year-old subjects and found that 40.7% of men and 23.8% of women had clinical acne but smoking was not significantly associated with acne (odds ratio: 0.54, 95% confidence interval: 0.17–1.78). Although we used a different approach, a case-control design, reached a similar conclusion.

On the other hand, Schafer et al [2] examined 896 persons, aged 1–87 years, in a cross-sectional study and found the overall prevalence of acne was 26.8%. Acne prevalence was significantly higher in active smokers as compared with non-smokers (40.8% vs. 25.2%, odds ratio: 2.04, 95% confidence interval: 1.40–2.99). This study has a wide age range and if we consider the patients with the age range of our study (15–40 years), no association between acne and smoking could be found (54/102 smokers as compared with 93/184 non-smokers had acne, odds ratio:1.10, 95% confidence interval: 0.68–1.79, p > 0.05).

Smoking has been associated with several adverse effects on the skin [6]. But protective effects of smoking against certain inflammatory diseases, such as ulcerative colitis [7], acne rosacea [8], pemphigus vulgaris [9], and recurrent aphthous stomatitis [10] have also been reported. Available data do not support any association between acne vulgaris and smoking.

## Competing interests

The author(s) declare that they have no competing interests.

## Authors' contributions

AF participated in the design and conduct of the study and preparation of the manuscript.

RS participated in the design and statistical analysis of the study.

SMD participated in the design and conduct of the study.

MNK participated in the design and conduct of the study.

All authors read and approved the manuscript.

## Pre-publication history

The pre-publication history for this paper can be accessed here:


